# Development of accreditation standards for midwifery clinical education in Iran

**DOI:** 10.1186/s12909-022-03823-8

**Published:** 2022-11-01

**Authors:** Sara Abedian, Mojgan Javadnoori, Simin Montazeri, Shahla Khosravi, Abbas Ebadi, Roshan Nikbakht

**Affiliations:** 1 Midwifery Department, School of Nursing and Midwifery, Ahvaz Jundishapur University of Medical Sciences, Ahvaz, Iran; 2 Reproductive Health Promotion Research Center, Department of Midwifery, School of Nursing and Midwifery, Ahvaz Jundishapur University of Medical Sciences, Ahvaz, Iran; 3 Department of Community Medicine, School of Medicine, Tehran University of Medical Sciences, Tehran, Iran; 4 Behavioral Sciences Research Center, Life Style Institute, Baqiyatallah University of Medical Sciences, Teheran, Iran; 5 Nursing Faculty, Baqiyatallah University of Medical Sciences, Teheran, Iran; 6 Fertility Infertility and Perinatology Research Center, Ahvaz Jundishapur University of Medical Sciences, Ahvaz, Iran

**Keywords:** Accreditation standards, Accreditation program, Clinical education, Midwifery

## Abstract

**Background:**

Accreditation is one of the most important methods of quality assurance and improvement in medical education. In Iran, there are no specific midwifery education accreditation standards. This study was designed to develop accreditation standards for midwifery clinical education in Iran.

**Methods:**

This study was performed in Iran in 2021. It consisted of two phases. In the first phase, accreditation standards for midwifery education in the United Kingdom, the United States, Australia and the International Confederation of Midwives were thoroughly examined through a narrative review. The domains obtained from this phase were used as a framework for coding in the second phase. In the second phase, a qualitative study was conducted with a directed content analysis approach to determine standards and criteria for clinical midwifery education accreditation in Iran. Participants were policymakers and senior managers of midwifery education, faculty members of midwifery departments with clinical teaching experience, and final year undergraduate midwifery students. The participants were selected by purposive sampling method, and data collection continued until data saturation.

**Results:**

The standards and accreditation criteria of midwifery education from the review study were formed 6 domains: Mission and goals; Curricula; Clinical instructors; Students, Clinical setting; and Assessment. In the second phase, data analysis led to the extraction of 131 codes, which were divided into 35 sub-subcategories, 15 sub-categories, and 6 main categories.

**Conclusion:**

Implementing the specific and localized standards of clinical midwifery education in Iran can lead to improved quality of clinical education programs.

## Introduction

Clinical education is a dynamic process in which students gradually gain experience by attending the patient's bedside and putting into practice the concepts they have already learned in interaction with the instructor and the clinical setting [1]. The quality of community health care depends on the quality of education in the clinical environment, and quality clinical education leads to the graduation of more successful and competent students [2].

Understanding the problems of clinical education is the first step to improving its quality [3]. Various studies have been conducted to evaluate the quality of clinical education, and in most of these studies, the quality of clinical education has not been at a desirable level [4, 5]. Given the paramount importance of midwifery clinical education in the health of the community, low-quality midwifery education leads to low-quality maternal and newborn health. Delivering high-quality midwifery care requires a professional, qualified and competent workforce. Improving the quality of clinical services plays an important role in the health system, and the government's policy is first to improve the quality of clinical education, and then, to improve the quality of health services [6].

Regulatory agencies must ensure that institutions meet the necessary qualifications for quality education, and this entails accreditation of educational programs [7], which is regarded and implemented as an important strategy to improve the quality of midwifery care [8].

Accreditation in healthcare settings is a common strategy to improve healthcare standards [9] and is one of the most important methods of quality assurance and improvement in medical education [10]. Accreditation refers to the process of quality control and assurance in higher education that allows an institution or its programs to be reviewed and certified to ensure that the required standards are met so that the institution is recognized and certified [11]. An accreditation system of educational programs aims to improve the academic performance and educational programs of an educational institution and then to increase the quality of care for its graduates [12]. By improving the learning environment, accreditation can also enhance the students' acquisition of skills in that environment [13]. Implementing a credible, comprehensive, and effective accreditation program has been reported to be a way to ensure quality and responsive midwifery education [14]. In accreditation, the development of standards is essential because these standards are the actual guidelines with which universities should comply. Not only are standards the basis for judgment but they are also formulated based on rules and according to the opinion of competent individuals. Therefore, standards can be considered as a measure or scale for judgment [15]. How to achieve standards varies from country to country, and there is no unique way to set standards. In compiling standards for an educational setting, it is smart to rely on scientific findings, experts’ opinions, the philosophy and goals of the educational system, facilities of the educational system, programs and policies of the educational system, and the laws and regulations governing the country [16]. For them to be enforceable, standards must be following the political system, executive structure, and legal duties of higher education institutions in each country. For this reason, accreditation standards can in no way be a translation of the standards of other countries [17]. Among pioneers in midwifery education, the efforts of the United States [18], Australia [19], and the United Kingdom [20] in developing midwifery education standards are noteworthy. The ICM has also set global standards for midwifery education to provide optimal care [21].

The training of competent midwives who are qualified to provide quality midwifery services requires the management and organization of midwifery educational environments, effective governance, adequate educational resources, and the establishment and extension of clinical simulation centers for continuous education. However, midwifery institutions in many low- and middle-income countries face many challenges in providing quality learning and teaching activities [22]. Although worldwide educational and international standards for the quality of midwifery education have been defined, a wide variety is readily observed in the type and nature of midwifery education programs in different cultures [8]. Improving the quality of clinical education leads to the training of competent and expert personnel [23]. Therefore, it is necessary to pay serious attention to the standards and criteria of effective (pre-service) clinical education in midwifery accreditation. The first step in establishing accredited clinical midwifery education is to develop accreditation standards tailored to midwifery education.

In Iran, medical universities are affiliated with Iran’s Ministry of Health and Medical Education, whereas non-medical universities are under the direct supervision of Iran's Ministry of Science, Research, and Technology. The Ministry of Health and Medical Education in Iran delegates the training of midwives to Schools of Nursing and Midwifery that are part of medical universities. Midwifery can be studied in public and private universities in Iran. Public universities are affiliated with the Ministry of Health while private universities (Islamic Azad University) are only under the periodic supervision of this ministry. In Iran, it is possible to study midwifery up to a Ph.D. level. Midwifery certificate to practice may be sought as an associate degree (2-year degree), direct entry in Bachelor’s degree (4-year degree), or discontinuous Bachelor’s degree (completing 2 extra years of studies after acquiring the associate degree in midwifery). In direct entry into midwifery, students must pass 130 credits (both theoretical and clinical) to gain a Bachelor’s degree. In Iran, educational quality assessment for universities and educational institutions is managed by the government [24].

There are no specific accreditation standards for midwifery education. Instead, the same general accreditation standards are used for all medical sciences programs. Given the lack of any specific local standards for the accreditation of midwifery clinical training, research in this field seems to be a much-needed line of inquiry. Therefore, the members of the present research team took advantage of the global achievements and experiences of Iranian experts in this field to develop accreditation standards for midwifery clinical education in Iran. Thus, the research question that this study sought to answer was: What are the domains, standards, and criteria for the accreditation of midwifery clinical education in Iran?

## Material &methods

This report is part of a broad project as a PhD thesis in 2021 aimed to develop clinical midwifery education accreditation standards in Iran.

In the first phase, the accreditation standards of midwifery education were examined through a narrative review. For this purpose, we reviewed the literature on accreditation and its methods as well as the accreditation standards for midwifery education at an international level. The United Kingdom, the United States, Australia, and the ICM, which are pioneers in the field of accreditation, were selected as targets.

Inclusion criteria were program accreditation standards for midwifery undergraduates, English scholarly articles on this topic, and relevant valid documents. Publications such as books, hospital accreditation standards, and postgraduate accreditation standards were excluded from the study.

In the second phase, a qualitative study was conducted with a directed content analysis approach to explore the standards and criteria for midwifery education accreditation standards. In this phase, the domains extracted from the first phase (narrative review) were used as a framework for formulation of study question and coding process The participants were members of the Midwifery and Reproductive Health Board in ministry of health and medical education in Iran, policymakers and senior managers of midwifery education and services in Iran, faculty members of midwifery departments with clinical teaching experience, clinical midwifery instructors, and final year undergraduate midwifery students. Participants were selected by purposive sampling method, and data collection continued until saturation [25]. The inclusion criteria were willingness to participate in the project and the ability to provide rich and sufficient information on the subject of the study. Failure to attend the interview session was one of the exclusion criteria. Written informed consent was obtained from all participants.

Data were collected through semi-structured individual interviews. The interview questions were prepared and used by the interviewer in a semi-structured manner based on the interview process in six areas of: “Mission and goals”; “Curriculum”; “Clinical instructors”; “Students”, “Clinical setting”; and “Assessment”. Then, according to the responses, follow-up and exploratory questions were asked to give them a deeper perspective. These included questions such as “Why?”, “How?”, “Could you give more explanations in this regard?” or “What are the features of your desired criteria?” After obtaining written informed consent from the participants, the interviews were recorded and transcribed verbatim for analysis [26]. Paralinguistic data such as tone of voice and facial expressions were recorded on a sheet that included the time and place of the interview. To increase the accuracy of the collected data, the researcher listened to the interviews repeatedly. The method of template-directed (inductive) content analysis presented by Elo & Kyngäs was used for data analysis. Content analysis under this framework involves three phases preparation, organization, and reporting [27].

Afterward, the whole transcription of the interview was read verbatim. Based on their semantic similarity, the codes were placed in pre-identified categories, and depending on the breadth and logical relationship of the data in categories, sub-categories were formed. Guba and Lincoln's criteria were also used to evaluate and ensure the accuracy and validity of the findings [28]. To make sure of trustworthiness, a sample of the interview transcription was reviewed and verified by the participants including faculty members and a graduate midwifery student (member check). To confirm the credibility of the study, about 50 percent of the process of data analysis was examined by two researchers in the field of midwifery with a background in qualitative research (Peer check) and prolonged engagement in the research topic (Prolonged engagement). Data transferability was achieved through maximum variety in the selection of participants and detailed description of the study for the sake of transparency of the study. As far as the dependability of the study was concerned, continuous comparative analysis of data and triangulation of data sources through interviews with key and subsidiary participants were performed. Finally, to ensure the confirmability of the study, the documents were recorded over time. In the process of coding and categorization, methodological consistency, external check, and peer debriefing were taken into account.

Finally, the obtained standards and criteria of midwifery clinical education accreditation were entered the Delphi phase for validation. However, due to the large amount of information, it was not possible to present the findings in this article, but they will be presented in a separate article.

The study was approved by research ethics committee of Ahvaz Jundishapur University of Medical Sciences, Ahvaz, southwest of Iran (Ref. ID: IR.AJUMS.REC.1400.126). To comply with ethical principles in research, written informed consent was obtained from the participants, and they were assured of the confidentiality of the information and accurate presentation of data.

## Results

### First phase

In the review study, the documents related to the accreditation standards of midwifery education in the United States, the United Kingdom, Australia, and the ICM were examined (Table 1).

**Table 1:** The comparison of the domain titles /the accreditation standards in the selected countries

Domain Titles	Organization / Mission	Curricula	Faculty	Student	Assessment Strategies	Resources, Facilities	Management of midwifery practice experience	Complaints and grievance
**Country/Organization**								
**ICM** (ICM 2017)	×	×	×	×	×	×	O	O
**The United States** (MEAC 2020)	×	×	×	×	O	×	O	×
**The United Kingdom** (NMC 2018)	O	×	×	×	O	-	-	-
**Australia** (ANMAC 2014)	O	×	-	×	O	×	×	-

× The country includes the same domain title (or is very similar)

O The country has partial overlap with the domain title

- The country does not have the same domain title or partial overlap

The results show that the standards of accreditation for midwifery education are different in different countries. For a comparison of different domains, the accreditation standards in selected countries are presented in Table 1.

The standards and criteria of the selected countries were thoroughly reviewed by the research team several times. Based on this, a set of criteria and sub-criteria of each country was obtained to be used for comparison. The final review was performed by the research team through repeated examinations of clinical accreditation standards in midwifery education in the selected countries. Finally, after making the necessary corrections and modifying categorization according to the midwifery curriculum and job description of midwives in Iran, the main categories (domains) were extracted. The results of extracting accreditation standards and criteria for midwifery schools were divided into 6 areas: "Mission and goals"; "Curricula"; "Clinical instructors"; "Students", "Clinical setting"; and "Assessment".

### Second phase

Participants' characteristics are presented in Table 2 (*n* = 15). The participants ranged in age from 22 to 56 years. There were 3 undergraduate students, 3 postgraduates, and 9 faculty members holding doctoral degrees.

**Table 2:** Characteristics of participants in the second phase of the study (interviews)

No	Age	Educational Degree	Occupation/Position	Work experience (years)
1	48	Ph.D. in Reproductive Health	A faculty member of the midwifery department/ Member of the Midwifery Board	22
2	45	Ph.D. in Medical Education	A faculty member of the department of medical education	19
3	54	Ph.D. in Reproductive Health	A faculty member of the midwifery department/ Member of the Midwifery Board/ Advisor of the Minister of Health	28
4	22	Undergraduate student of Midwifery	Final year student	-
5	22	Undergraduate student of Midwifery	Final year student	-
6	23	Undergraduate student of Midwifery	Final year student	-
7	55	Ph.D. in Reproductive Health	A faculty member of the midwifery department/ Member of the Midwifery Board/ Head of the midwifery department	25
8	43	Ph.D. in Reproductive Health	A faculty member of the midwifery department/ Member of the Midwifery Board	10
9	56	Ph.D. in Reproductive Health	A faculty member of the midwifery department/ Member of the Midwifery Board/ Head of the midwifery department	28
10	47	Master’s degree in Midwifery	A faculty member of the midwifery department/Dean	22
11	48	Ph.D. in Reproductive Health	A faculty member of the midwifery department	23
12	33	Master’s degree in Midwifery	Clinical Instructor of midwifery department	5
13	56	Ph.D. in Midwifery	A faculty member of the midwifery department	19
14	31	Master’s degree in Midwifery	Clinical Instructor of midwifery department	9
15	58	Ph.D. in Reproductive Health	A faculty member of the midwifery department	32

The domains extracted from the review study in the first phase were used as a guide in formulating questions and conducting semi-structured interviews in this phase. Interview questions were prepared and posed by the interviewer in a semi-structured manner based on the domains of "Mission and goals"; "Curricula"; "Clinical instructors"; "Students", "Clinical setting"; and "Assessment". Data analysis in this phase led to the extraction of 131 codes (sub criteria), which were divided into 35 sub-subcategories (criteria), 15 sub-categories (standards), and 6 main categories (Domains) (Table 3).

**Table 3:** Analysis of categories, subcategories, and sub-subcategories for accreditation standards of clinical midwifery education

Main Category (Domains)	Subcategory (standards)	Sub-subcategory (Criteria)
Mission and goals	Objectives of clinical education	Specific objectives of clinical education
		Development and review of clinical education goals
	Clarity of mission	Educational mission
		Service mission
Curricula	Curriculum	Clinical education curriculum content
		Accurate implementation of the curriculum
		Hidden curriculum
		Educational strategies
		Comprehensive center for skill lab training
	Virtual clinical education	Clinical education in a non-clinical setting
		The necessary infrastructure for virtual education
Clinical instructors	Skills of clinical instructors	Teaching-learning skills
		Clinical skills
		Communication skills
	Empowerment of clinical instructors	Continuation of training of clinical instructors
		Support for clinical instructors
	Collaboration between hospitals and the faculty in terms of clinical education	Collaboration of faculty members
		Collaboration of clinical staff
Students	Requirements for starting clinical education	Assessing the scientific status of students
		Preparation of students for the clinical setting
	Educational services for students	Identification of learning problems
		Handling complaints and criticisms
Clinical setting	Educational conditions in clinical settings	The comprehensiveness of clinical setting
		The match between the internship program and the clinical setting
	Clinical staff	Clinical staff’s acceptance of student education
		Collaboration of clinical staff with student education
	Clinical facilities and equipment	Educational assistance facilities
		Clinical facilities for students
		Clinical facilities for clinical instructors
Assessment	Students assessment	Assessment of students
		Assessment of topics
	Assessment of clinical instructors	Assessment of clinical instructors
		Assessment of feedback
	Assessment of clinical settings	Assessment of physical space
		Process assessment

### 1- Mission and goals

This category involves creating the desired vision and perspective by managers with the participation of faculty members and related groups. It also includes agreement on the mission and leading goals according to which educational institutions can fulfill their mission and achieve the desired vision. The participants in this study emphasized this category as one of the accreditation standards in midwifery clinical education. This category has the following two sub-categories.

#### Objectives of clinical education

The participants insisted that the goals of clinical education be based on the curriculum, the needs of the community, and the prevailing culture of that community.

“Clinical midwifery education is valid as far as its goals are correctly defined. I mean, it should be according to the curriculum and the needs of society and should have the right strategy to achieve these goals” (P10).

The midwives recommended the formation of a working group to review the goals of clinical education and, if necessary, modify it as needed.

“We should have a working group to review and modify the objectives of clinical education from time to time according to the needs of the community, if necessary” (P8).

#### Clarity of mission

For the participants, it was a fundamental principle to educate capable students who are competent for clinical and professional ethics. Such students, they believed, are diligent in maintaining and promoting the health of the community under different circumstances including crises.

“An academic department should always know that its main mission is to train capable students” (P3). “In the midwifery department, we must seek to train students who can demonstrate their unique abilities during critical situations” (P11).

### 2- Curricula

Curriculum, which was appreciably emphasized by the participants in the research, is defined as any activity or activities that include achieving productivity, skills, and knowledge. This category consists of two sub-categories.

#### Curriculum

The participants believed that clinically teachable content should be specified in the curriculum.

“The content of the clinical education curriculum must be clear” (P10).

The midwives who participated in the study acknowledged that a lesson plan must be precisely derived from the curriculum.

“When I am writing a lesson plan, the alignment between the curriculum and the lesson plan is very important” (P15).

According to the participants, the alignment of the contents of their logbooks with the content of the curriculum and careful monitoring of the completion of these logbooks are of paramount importance.

“The content of the logbook should be consistent with the content of the curriculum, and the instructor should oversee the completion of these logbooks” (P3).

Most of the midwives participating in this study also placed enormous emphasis on the use and inclusion of hidden curriculum topics in the student curriculum.

“Whatever a midwifery student should be taught, I believe, should be included in the curriculum. It should be made clear in the curriculum how to behave professionally and ethically, how to communicate well with people, how to be fair in dealing with others, etc. When there is enough information and up-to-date books in this field, and we have excellent experts in this field, why not show it in the curriculum!” (P1).

However, some of the interviewed midwives had an opposing view: "*it is not possible to write the hidden curriculum in the open curriculum. As the name of the hidden curriculum suggests, it must be hidden and hidden in the teachers' behavior*" (P7).

Despite these conflicting views, the sub-criterion “attention to hidden curriculum topics in students' educational program” was considered for the criterion of the hidden curriculum. This was because of the importance of the topic of the hidden curriculum in the education of students according to the opinion of the research team.

The participants emphasized the need for clear clinical training methods and the use of evidence-based education.

“Clinical teaching methods should be very clear in the curriculum” (P3). “Evidence-based education is very important. Everything is not based on experience. I mean, the education you give and the action that is taken should be based on scientific articles and texts.” (P8).

According to the participants, it is very important that a codified program be there for using the skill lab and that the first educational procedures be performed by students in these centers.

“In the midwifery department, a part of the training, especially the initial part, should be offered in the skill lab” (P12).

#### Virtual clinical education

According to the participants in this study, the use of distance education along with in-person clinical education is fruitful provided that there are appropriate facilities and equipment.

“I believe in distance education. If anything, clinical education should not necessarily be at the patient's bedside” (P13).

“Telemedicine and distance assessment has a long history in the world. I have a positive opinion about distance education. This virtual education should not be limited to pandemic conditions. Some clinical courses should be offered through this modality, but by meeting the requisites first. Finally, its value should be equal to or even higher than in-person education”. (P10).

### 3- Clinical instructors

In clinical education, the presence of a good clinical instructor is very important due to the sensitivity of the health of the community. This category is very critical in clinical research on midwifery education. It consists of 3 sub-categories.

#### Skills of clinical instructors

The participants in the study believed that clinical instructors must possess the knowledge of up-to-date scientific procedures and the ability to offer clinical education.

“A clinical instructor should be aware of the latest scientific achievements in the treatment of patients and be able to offer proper education to the student”. (P1).

The participants deemed clinical work experience very crucial for clinical instructors and placed immense emphasis on this experience.

“We should not allow anyone to come and teach students in clinical settings! They must have at least a few years of experience in that field and be interested in teaching students” (P2).

The interviewees in the study accentuated clinical instructors' communication skills including communication with patients, students, etc., and considered them role models for students.

“There are some professors who do not treat the patient well, and the student learns this because we do not learn it from somewhere else! The students follow the example of their professors!” (P6). “In my opinion, when choosing a professor, the way they communicate with students, patients, and the hospital staff should be taken into account. Simply having a degree or qualification is not enough.” (P14).

#### Empowerment of clinical instructors

According to the participants in this study, clinical instructors should be trained in various fields such as teaching methods and clinical assessment.

“Unfortunately, many clinical instructors do not pay attention to clinical education and assessment! Clinical educators need to be fully trained in this area”. (P8).

The midwives who participated in the study maintained that legal support for clinical instructors in internship programs leads to better education for students.

“The clinical instructor should be legally supported so that if something happens, they will not be held responsible for it. This support will increase the instructor's interest in doing clinical work with the student”. (P12).

#### Collaboration between hospitals and the faculty in terms of clinical education

The participants attached great importance to the collaboration of faculty members and clinical staff when it came to the student's clinical education.

“To teach educational content, it is better if a part of it is taught by a faculty member and the other part is left to a person who works in a clinical setting (i.e., the staff). This will increase the effectiveness of the training.” (P3).

### 4- Students

Students are individuals for whose scientific and practical advancement proper planning should be done so that they are prepared to provide services to the needs of society. This category consists of two sub-categories.

#### Requirements for starting clinical education

The participants believed that it is useful to check the students’ readiness before starting clinical education and recommended that, if necessary, the student's competence should be strengthened by methods such as holding workshops, etc. Of course, this should be following the goals of the internship programs.

“In my opinion, before the students enter the internship programs, their readiness should be checked and, if necessary, boost it in different ways.” (P8).

According to the participants in the study, a package to familiarize students with the clinical field and the rules governing the wards is useful for the success of clinical education.

“The student must be familiar with the environment before entering the clinical field. A good clinical education has a ready-made package for this purpose, and the student must be familiar with it before going to the clinical field. This means learning the ropes: whom they are in contact with, what the scope of their authority and duties in that ward is, and with what purpose they have entered that ward.” (P10).

#### Educational services for students

Participants in the study stated that a clinical counselor who can identify the educational problems of students in the clinical field and who is willing to address these problems is needed.

“It is good to have someone as a clinical counselor to identify the educational problems of students in clinical settings and try to solve those problems with the cooperation of faculty officials.” (P15).

### 5- Clinical setting

A clinical setting is an ideal environment for teaching and learning and lies at the heart of clinical midwifery education. This category consists of three sub-categories.

#### Educational conditions in clinical settings

As far as clinical settings were concerned, the participants believed that a relevant number of students and patients referring to a particular clinical center is important.

“One of the important points about clinical settings is the ratio of the number of students to a particular clinical field, and another is how general that field is. In some general centers, students need to see some rare diseases at least once”. (P9).

The participants also insisted that the suitability of the clinical setting to implement the relevant curriculum is useful in educating students.

“The clinical setting must meet the necessary conditions to implement that curriculum.” (P3).

#### Clinical staff

Participants in this study stressed that how the clinical staff accepts the presence of students and their instructors in the clinical setting is very important.

“Staff in hospitals, even some university hospitals, are not briefed on cooperating with the instructor and the student. The staff should be informed that students need to do a lot of things and that they have to treat the students more patiently” (P12).

#### Clinical facilities and equipment

The participants strongly recommended the provision of educational facilities such as conference rooms, resourceful libraries with up-to-date books, computers with an Internet connection, and skill labs with appropriate facilities to meet the educational needs of students.

“Students in the clinical setting also have a series of educational needs such as what they should do when there is no patient. There should be a series of educational assistance facilities such as the Internet, a library, and a skill lab to empower the student”. (P10).

The midwives participating in this study found it necessary to provide clinical facilities for students and clinical instructors.

“There should be a pavilion in the hospital for my students. I expect my students to work night shifts but I do not expect them to stay up all 12 h! The instructors must also have a suitable pavilion.” (P7).

### 6- Assessment

Improving the quality of clinical education requires continuous assessment of its current status, identification of its strengths, and alleviation of its weaknesses. This category consists of three sub-categories.

#### Student assessment

The participants laid particular emphasis on how the students were assessed.

“Student assessment should be continuous. In every internship program and every session, a score should be assigned to any student activity, and the students should evaluate themselves at the end of each session”. (P12).

According to the participants in the study, student assessment includes topics such as practical skills, communication skills, professional ethics, and teamwork.

“In addition to evaluating students’ practical and communication skills, another thing that should be evaluated, which is not, is teamwork. Our students must learn how to work together”. (P2).

#### Assessment of clinical instructors

The students participating in this study valued the importance of faculty assessment feedback.

“In my opinion, assessment is worth that be affected and follow up if a professor has a weakness in a field she corrected herself or not?!” (P5).

#### Assessment of clinical settings

As far as the assessment of clinical settings was concerned, the participants in the study pointed out that what is of particular importance is a provision of an appropriate place for the students to reside and another for educating them.

“Internship programs should be offered in a well-prepared area for students to attend, work and train”. (P14).

Healthcare facilities should be evaluated in terms of the variety of patient coverage and patient care according to the guidelines of the Ministry of Health.

“It is better to check the number of referrals in the hospital intended for student internship programs. Do all referrals range from low-risk to high-risk? Are they from all socio-economic classes in society? Do the hospitals take care of patients according to the clinical guidelines of the Ministry of Health?” All these affect the students’ educability”. (P10).

## Discussion

This study aimed to develop accreditation standards for clinical midwifery education in Iran. The standards of accreditation of clinical midwifery education in Iran were organized into the following categories: “Mission and goals”; “Curricula”; “Clinical instructors”; “Students”, “Clinical setting”; and “Assessment”. These standards will serve as a prerequisite to ensure that midwifery students achieve the desired learning outcomes in the clinical curriculum and become an effective workforce.

The domains of “Mission and goals”, “Curricula”, “Clinical instructors”, “Students”, and “Assessment” were almost present in most of the studied countries' accreditation standards. However, the domain of "Clinical setting" did not exist separately in any of the investigated countries. Nonetheless, there are partial references to Clinical settings in these countries. In the U.S, for example, this could be traced in accreditation standards for midwifery education regarding "Facilities, Equipment, and other Resources". In Australia, this is dealt with under "Management of midwifery practice experience". ICM has "Assessment strategies" and "resources, facilities, and services" for this purpose. Due to the importance of the subject and the fact that in the present research the focus is on the accreditation standards of midwifery education in the clinical setting, the field of "Clinical setting" was placed as a separate field in the accreditation list of midwifery clinical education. This allows a more detailed examination and monitoring of the clinical areas.

In order for students to achieve the desired learning outcomes, it is necessary to receive effective training in "Clinical settings". In this regard, Renfrew et al. acknowledge that if the midwifery staff is trained in a supportive clinical environment, they will be well prepared to provide quality midwifery care [29]. In one study, the inclusion of separate clinical sites was recommended in the accreditation process of midwifery schools [30]. In the present study, one of the axes of accreditation of clinical midwifery education was the clinical setting.

The current literature repeatedly points out that the use of accreditation standards is a fundamental principle to guarantee education and achieve educational goals [31, 32]. Not until a country can validate midwifery education, will it ensure high-quality education [33].

Given the fact that less than half of the countries in the world have accreditation systems for midwifery education, the use of ICM standards is recommended to develop national standards for every country [34]. The recent unprecedented surge in the number of migrations of health professionals in Iran [35] has led to calls for international oversight of key elements of health workers’ training, including accreditation of education [36], so that destination countries can ensure that migrant health professionals have the necessary qualifications. This will make it easier for these professionals to make informed choices about where to work and to go to the place where they are needed. The use of accredited standards of developed countries and ICM in midwifery education provides an opportunity to harmonize educational standards. In order to support midwifery students and ensure that graduates have used international ICM standards for midwifery education, in the first phase of the present study, the standards of developed countries and ICM were used as a framework and guide.

The domain of "Mission and goals" is the basis of midwifery education accreditation standards among other domains. It is necessary to set the correct mission, goals, and planning to meet the desired demands and guarantee quality. In line with the obtained results, Ann considers having planning, especially long-term and short-term planning, essential for success in the accreditation process [37]. The goals, mission, and strategic plan of the midwifery profession in each society should be based on the needs identified through a comprehensive examination of the conditions of the faculties and in accordance with the executive structure that governs the macro-educational policy of each region and country. In this research, the standards of the goals of clinical education and the clarity of the mission of the program were determined under "Mission and goals".

The “Curricula”, as expected, were the main domain in all accreditation programs. It is important to note that among the accreditation standards of the countries studied, only the United States mentioned “Virtual classrooms and distance learning” in the area of “Curriculum and resources, and facilities”. It seems that virtual education is very important in today's society. The importance of this educational method lies in its potential to save time, reduce costs, transform traditional education into modern learning, and increase efficiency. With the start of the Covid-19 epidemic around the world, virtual education has exerted a great impact on increasing the quality of the teaching–learning process. Therefore, this type of education is recommended to be included in accreditation programs. In this research, the use of virtual education in clinical education was highlighted as one of the standards in the domain of the curricula.

In the present study, within the Curriculum standard, the following points were emphasized: the criteria for choosing the content of clinical education curriculum, accurate implementation of curriculum, attention to the hidden curriculum, educational strategies, and a comprehensive center for clinical skills training.

Issues related to lack of clinical experience in midwifery education create a challenge in most countries, and this has been identified in the recent initiative of the World Health Organization to strengthen the quality of clinical midwifery education [38]. A previous study showed that there are a number of colleges that do not have approved clinical skill centers and that some of the instructors of these colleges do not have professional qualifications for clinical education [30]. According to the literature, the main challenges of clinical midwifery education have been incompetent professors, limited opportunities for the students to acquire the required skills, and a lack of facilities and equipment [39, 40]. Successful implementation of curricula calls for the presence of qualified and skilled professors [41]. The results of a systematic review showed that the need to update the knowledge and skills of instructors in clinical practice is essential [42]. In our study, the empowerment of clinical instructors, which was considered one of the standards within the domain of "Clinical instructors", is one way to realize the mission and goals of the department.

The domain of "Students" was among the common domains agreed upon by most countries as far as the midwifery education accreditation program was concerned. The greatest importance of accreditation lies in the learning outcomes of students who graduate from their training course, and the direct support of these students plays a significant role in achieving accreditation goals [43]. Adequate monitoring of student performance in clinical settings and creating appropriate opportunities for them to gain clinical experience are two essential requisites for obtaining essential midwifery qualifications [44]. These factors were included in the accreditation standards of clinical midwifery education in the present study.

In the current study, the domains of "Clinical instructors" and "Students" were stated as two important accreditation domains in midwifery clinical education.

The appropriate assessment of students, professors, and the clinical setting in educational accreditation can lead to the formation of a profile of the current state of the educational program, which in the next stages, can serve as a decision guide for planning, teaching professors, and improving the quality of education. Over time, these continuous assessments provide critical information that can be used for effective decision-making, performance control, and resource allocation [45].

According to McCarthy et al., the accreditation process and standards are different in different countries across the world, and it is recommended that specific accreditation standards be developed for each country with the help of experts in the field [46]. In addition to developing standards that are comprehensive and localized, involving stakeholders in the development of standards can help in the implementation of these accreditation standards. Accreditation standards enable institutions to witness continuous improvement in the quality of education. By abiding by these standards in education, we will continuously see improvement in academic performance and its consequent results in the health sector [47]. In the current research, the opinions of specialists in this field were also used to formulate the accreditation standards for midwifery clinical education.

Naturally, only after a specific approach is chosen in each country to develop and set accreditation standards, will the specific challenges of that community become apparent. In order to minimize the challenges ahead, more importance should be attached to the choice of accreditation standards. Due to differences in training levels and executive capabilities, the use of accreditation standards without localization of standards adopted from other countries- even developed ones- is not recommended. Given the ever-increasing expansion of knowledge, it is essential that governments, especially those of less developed countries, support the establishment of structures for the development of midwifery accreditation standards in line with global policies in order to facilitate the education of capable and competent graduates.

In the accreditation standards of other countries, however, the midwifery education program is evaluated as a whole. But the difference of the current research is that it evaluates the clinical education of midwifery students, which has a great role in their practical skills, in more detail.

In this study, standards were proposed to integrate clinical midwifery education and ensure the implementation of clinical education programs.

## Conclusion

Global efforts to improve the health of mothers and infants depend on a strong workforce in midwifery. This requires a quality education system that can train qualified midwives. Such changes are expected to appear in both the health system and the quality of the Iranian health system by enforcing accreditation standards for clinical midwifery education. This study provides a platform for other countries how to set standards for accreditation of clinical midwifery education in line with national priorities, which will lead to the development of policies integrated with global standards for clinical midwifery education. Finally, the clinical midwifery education standards enumerated in this article, which are based on international standards and have been widely reviewed by midwifery experts and educators, are used to guide instructors, policymakers, and governmental and non-governmental organizations.

## Data Availability

The datasets supporting the conclusions of this manuscript are included within the article.

## Abbreviations

ICM: International Confederation of Midwives
U.S: The United States
U.K: The United Kingdom
